# Supplementary material for: Diversity, Taxonomic Novelty, and Encoded Functions of Salar de Ascotán Microbiota, as Revealed by Metagenome-Assembled Genomes
Source: Microorganisms. 2023 Nov 20;11(11):2819. doi: 10.3390/microorganisms11112819 (PMC10673233; doi:10.3390/microorganisms11112819)
Supplement: Supplementary file 1 [file microorganisms-11-02819-s001.zip › Supplementary Material.pdf]

Diversity, taxonomic novelty, and encoded functions of Salar de Ascotán microbiota as revealed by metagenome-assembled genomes

Marcelo Veloso[1], Angie Waldisperg[1], Patricio Arros[1], Camilo Berríos-Pastén[1], Joaquín Acosta[1], Hazajem Colque[1], Macarena A. Varas[1,2], Miguel L. Allende[2], Luis H. Orellana[3], Andrés E. Marcoleta[1,*]

[1]Grupo de Microbiología Integrativa, Laboratorio de Biología Estructural y Molecular BEM Faculty of Science, Universidad de Chile. Las Palmeras 3425, Nuñoa, Santiago 7800003 , Chile.

[2]Millenium Institute Center for Genome Regulation. Faculty of Science, Universidad de Chile. Las Palmeras 3425, Ñuñoa, Santiago 7800003, Chile.

[3]Department of Molecular Ecology, Max Planck Institute for Marine Microbiology, Celsiusstr. 1, D-28359 Bremen, Germany.

[*]Correspondence: amarcoleta@uchile.cl

SUPPLEMENTARY MATERIAL

Contents of the Supplementary Material (this file and two spreadsheets):

Figure S1. Geographic location of Salar de Ascotán and the sampling sites.

Figure S2. Taxonomic assignment of the Illumina and Nanopore metagenomic reads.

Figure S3. Complete DRAM functional profile for archaeal and bacterial MAGs.

Figure S4. Functional profile of the Salar de Ascotán bacterial MAGs, according to the SUPER-FOCUS classification.

Figure S5. Functional profile of the Salar de Ascotán archaeal MAGs, according to the SUPER-FOCUS classification.

Table S1. Bacterial genera and their relative abundance in Salar de Ascotán water, sediment, and soil (provided as a separate spreadsheet).

Table S2. Sequencing and assembly stats of the Salar de Ascotán metagenomes.

Table S3. Main stats of the bacterial and archaeal MAGs recovered from Salar de Ascotán soil, sediment, and water (provided as a separate spreadsheet).

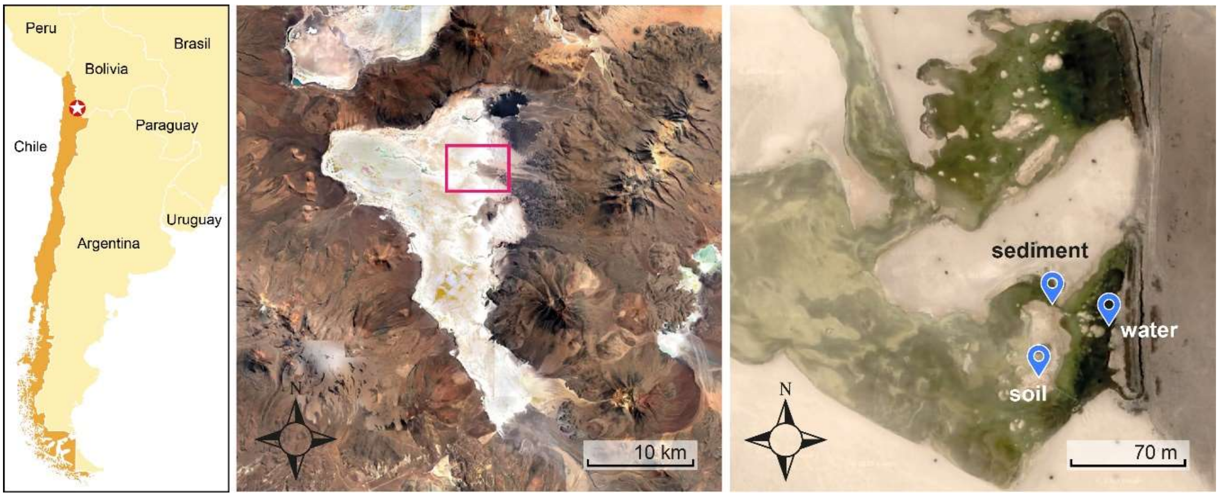

Figure S1. Geographic location of Salar de Ascotán and the sampling sites. The zoom of the view increases from the left to the right.

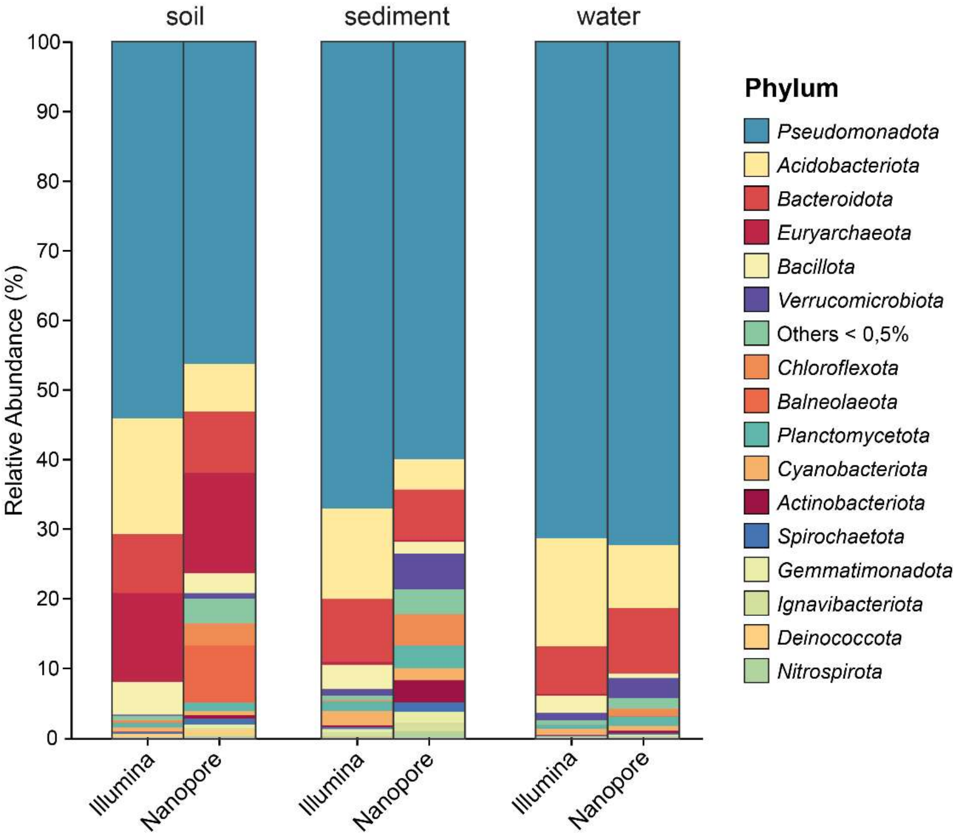

Figure S2. Taxonomic assignment of the Illumina and Nanopore metagenomic reads. The shotgun metagenomic reads obtained for each sample were classified using Kraken (Nanopore) and Kaiju (Illumina). The bars show the relative abundance of the reads classified at the phylum level. Phyla with abundances below 0.5% were grouped into the “others” category.

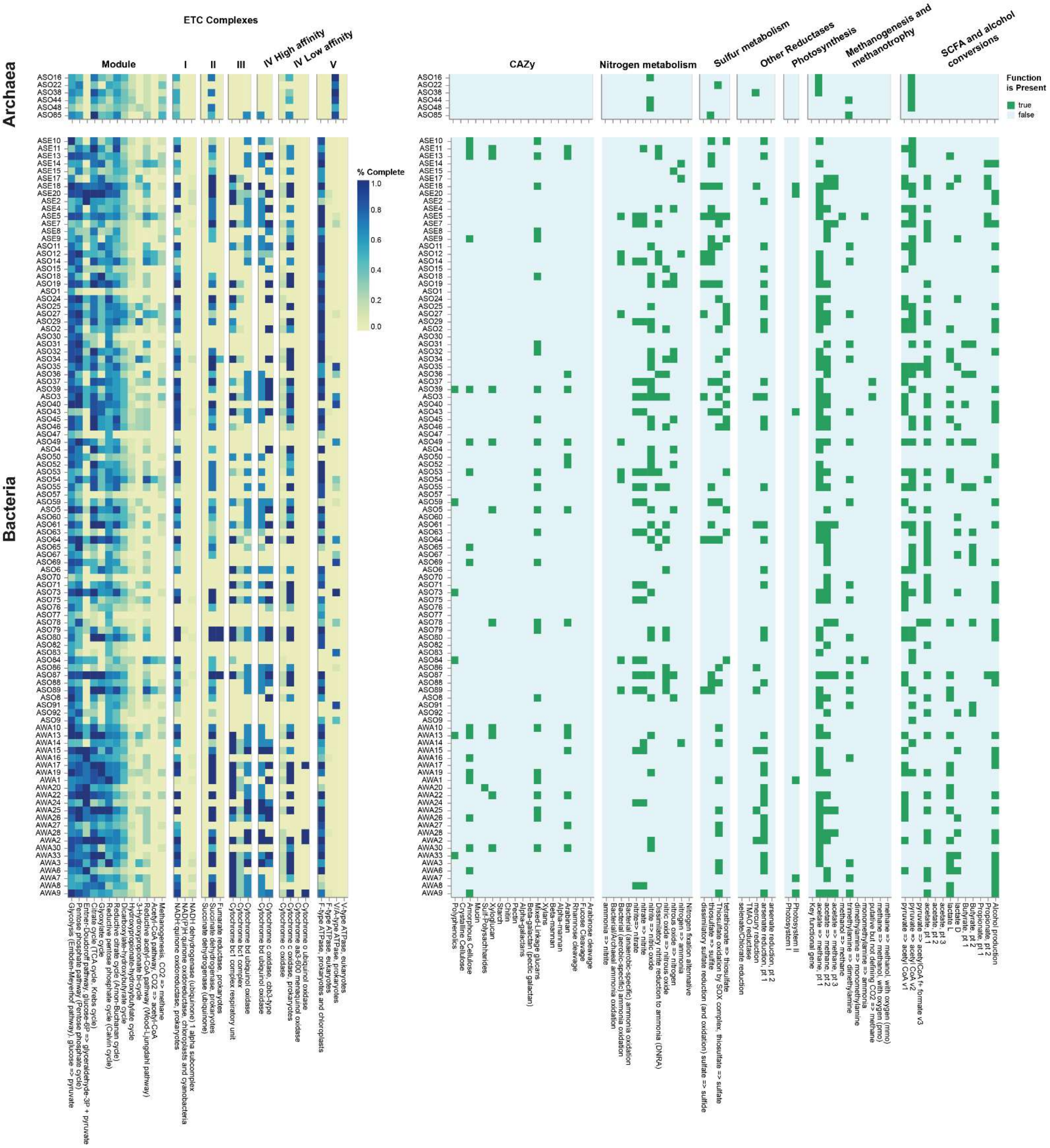

Figure S3. Complete DRAM functional profile for archaeal and bacterial MAGs. DRAM categorization of the metabolic pathways and molecular functions. Sections of the heatmap are ordered to highlight pathway completion (Module), subunit completion (Electron transport chain (ETC) complexes), and presence/absence of the genes required for the corresponding pathway step (panels to the right show).

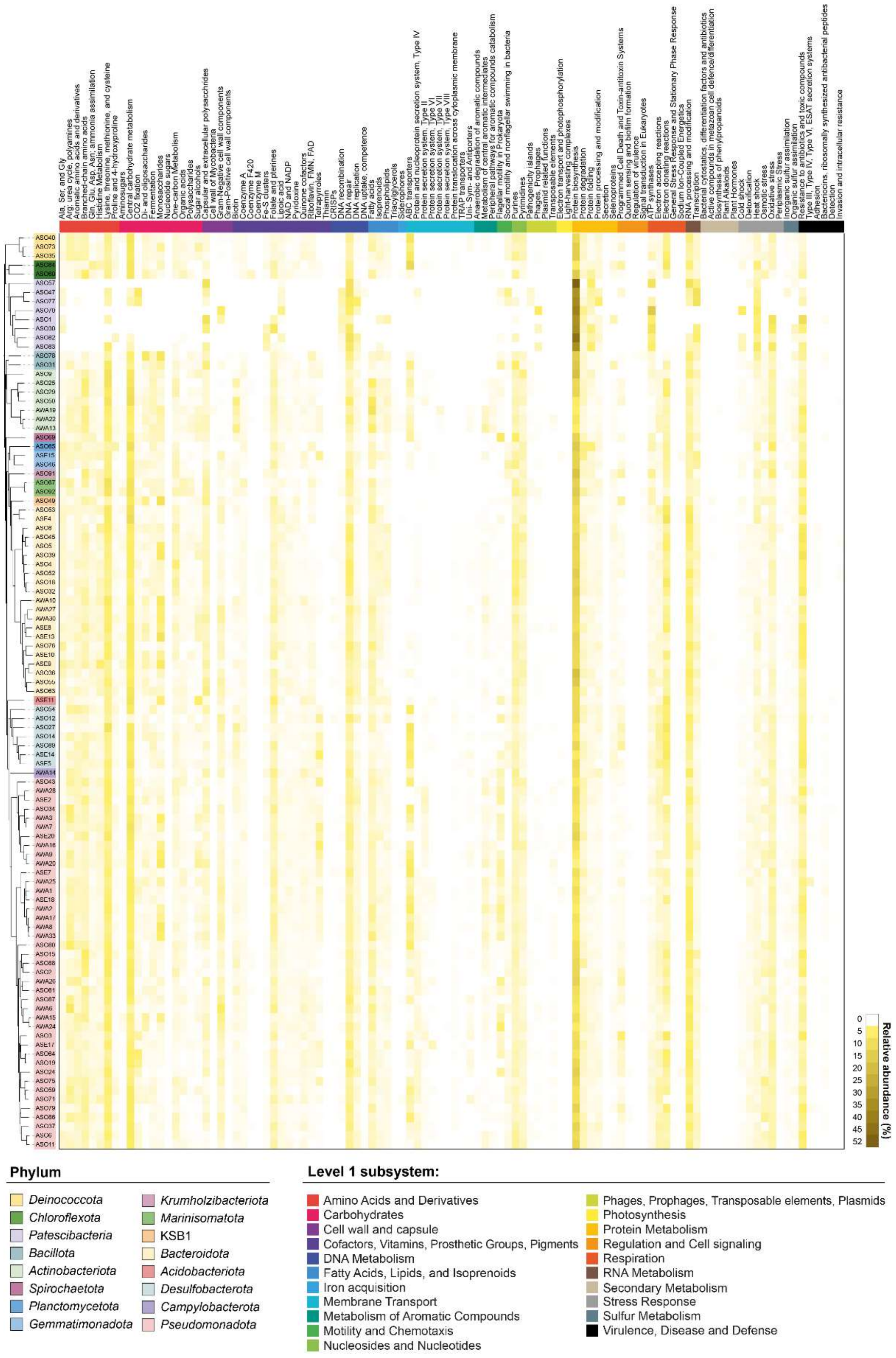

Figure S4. Functional profile of Salar de Ascotán bacterial MAGs, according to the SUPER-FOCUS classification in the hierarchical subsystem levels 1 and 2. The heatmap indicates each category's relative abundance among the total number of categories for each MAG.

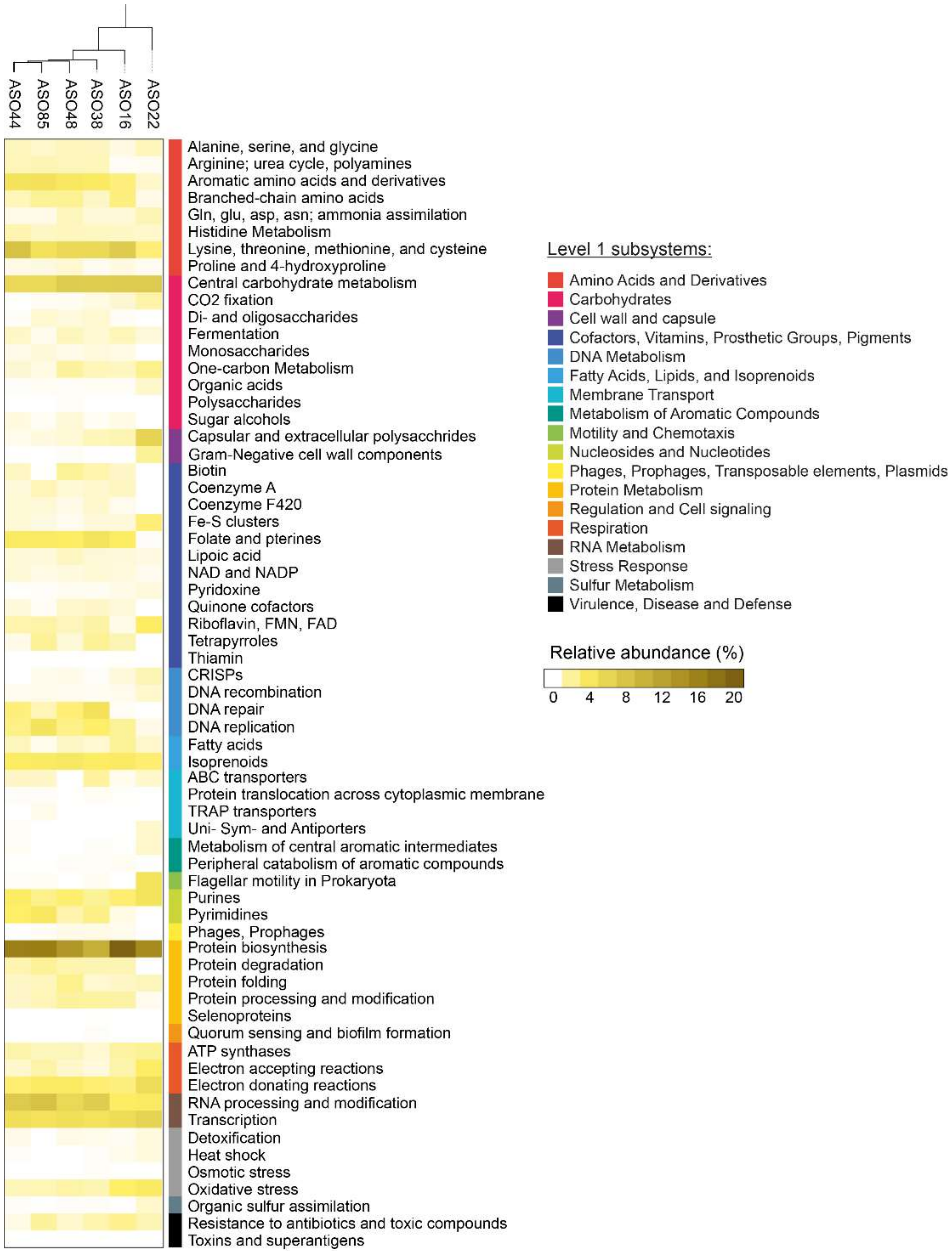

Figure S5. Functional profile of Salar de Ascotán archaeal MAGs, according to the SUPER-FOCUS classification in the hierarchical subsystem levels 1 and 2. The heatmap indicates each category's relative abundance among the total number of categories for each MAG.

Table S2. Sequencing and assembly stats of the Salar de Ascotán metagenomes.

Parameter	Sample		
	**Soil**	**Sediment**	**Water**
**Nanopore total bases**	9.77 Gbp	6.71 Gbp	9.49 Gbp
**Nanopore average read length**	6,518 bp	6,219 bp	3,742 bp
**Nanopore average read quality (Q)**	11.8	11.3	12.2
**Illumina total bases**	11.60 Gbp	12.62 Gbp	12.37 Gbp
**Illumina average read length**	151 bp	151 bp	151 bp
**Illumina average read quality (Q)**	33.2	31.5	30.7
**Total assembly length**	697 Mbp	292 Mbp	330 Mbp
**# assembled contigs**	12,152	7,514	16,370
**Largest contig**	5.2 Mbp	1.2 Mbp	2.4 Mbp
**GC content**	56.1 %	57.6%	57.0%
**N50**	135.4 Kbp	63.6 Kpb	39.6 Kbp
**L50**	1069	1182	1679
